# Multiple Receptor-Ligand Interactions Direct Tissue-Resident γδ T Cell Activation

**DOI:** 10.3389/fimmu.2014.00602

**Published:** 2014-11-24

**Authors:** Deborah. A. Witherden, Kevin Ramirez, Wendy L. Havran

**Affiliations:** ^1^Department of Immunology and Microbial Science, The Scripps Research Institute, La Jolla, CA, USA

**Keywords:** epithelial, γδ T cell, activation, costimulation, epidermis, skin

## Abstract

γδ T cells represent a major T cell population in epithelial tissues, such as skin, intestine, and lung, where they function in maintenance of the epithelium and provide a crucial first line defense against environmental and pathogenic insults. Despite their importance, the molecular mechanisms directing their activation and function have remained elusive. Epithelial-resident γδ T cells function through constant communication with neighboring cells, either via direct cell-to-cell contact or cell-to-matrix interactions. These intimate relationships allow γδ T cells to facilitate the maintenance of epithelial homeostasis, tissue repair following injury, inflammation, and protection from malignancy. Recent studies have identified a number of molecules involved in these complex interactions, under both homeostatic conditions, as well as following perturbation of these barrier tissues. These interactions are crucial to the timely production of cytokines, chemokines, growth factors, and extracellular matrix proteins for restoration of homeostasis. In this review, we discuss recent advances in understanding the mechanisms directing epithelial-T cell crosstalk and the distinct roles played by individual receptor-ligand pairs of cell surface molecules in this process.

## Introduction

Epithelial tissues represent barriers between the body and the outside world. These barrier tissues contain resident populations of T cells that help maintain homeostasis and provide a defense against disruption to the epithelium. One such T cell population is the γδ T cell. Subsets of γδ T cells are present in virtually all epithelial tissues of all species and, in many cases represent the major, or even exclusive, T cell population in the tissue (1). A variety of roles have been ascribed to these tissue-resident γδ T cells, including maintenance of epithelial homeostasis, tissue repair, inflammation, response to infection, and protection from malignancy (2–5). Thirty years have already passed since the discovery of γδ T cells and, although a considerable amount of progress has been made in the understanding of the varied functions of these cells, much remains unknown about the mechanisms by which these functions are elicited.

Like αβ T cells, γδ T cells express a rearranged T cell receptor (TCR), although with far more limited diversity than αβ T cells (1, 6). In contrast to αβ T cells, most epithelial-resident γδ T cells do not express the CD4 or CD8 coreceptors or the well characterized αβ T cell costimulatory molecule, CD28 (7, 8). This gave rise to the hypothesis that alternate molecules on γδ T cells may serve analogous functions to those well characterized as essential for αβ activation and that additional novel interactions may be responsible for some of the functions unique to epithelial γδ T cells.

Indeed, the intimate contact between γδ T cells and the neighboring epithelial cells they surveil, suggests that multiple receptor–ligand interactions likely maintain γδ T cells in their homeostatic state as well as participate in their activation and effector functions. This review will focus on recent advances in the identification and characterization of such molecules and the unique roles they play in epithelial γδ T cell function.

## Antigen Recognition

Stress-induced self-antigens have been postulated for many years to represent ligands for γδ T cells (7, 9). Although γδ T cell ligands are not the focus of this review, the γδ TCR forms an essential component of the cell’s ability to survive and function and the importance of TCR–ligand interactions for γδ T cell activation are undisputed. In some cases, bone fide ligands have been identified [reviewed in Ref. (10)]. Despite the restricted use of the γδ TCR, ligands appear to be varied and diverse in nature and the majority of those identified to date are ligands for circulating γδ T cells as opposed to the tissue-resident epithelial γδ T cells.

One of the populations of epithelial-resident γδ T cells that has received much attention, yet TCR-ligands remain unidentified, is the dendritic epidermal γδ T cell (DETC) of the murine epidermis (11, 12). These cells express an invariant Vγ3Vδ1 TCR [nomenclature according to Garman (13)]; alternative nomenclature Vγ5Vδ1 (14), that is expressed exclusively by DETC in skin and DETC precursors in fetal thymus (15). Recent work has demonstrated rapid and transient expression of the unknown TCR ligand following wounding, as well as a restricted distribution of expression to sites immediately adjacent to the wounds (16). In this study, no ligand was detectable under steady-state conditions in non-wounded tissue. In contrast, another study using intravital microscopy found constitutive Vγ3Vδ1 TCR signaling from interaction with neighboring epithelial cells, with wounding eliciting a reorganization of TCR molecules rather than an increase in signal strength (17). This suggested constitutive TCR–ligand interactions under homeostatic conditions. As neither study identified a TCR ligand, both lack definite proof of constitutive ligand absence or presence, respectively. The Skint1 molecule does represent an attractive candidate for a steady-state Vγ3Vδ1 TCR ligand, as it is constitutively expressed by keratinocytes (18). However, as yet, no direct binding of Skint1 to the Vγ3Vδ1 TCR has been demonstrated. Until the identity of the Vγ3Vδ1 TCR ligand is firmly established, it cannot be concluded that this constitutive signaling in DETC in the steady state is indeed ligand-induced. Nevertheless, Skint1 deficiency has a profound effect on DETC development (19, 20) and studies in Skint1-deficient animals have added to the body of evidence demonstrating the importance of the Vγ3Vδ1 TCR to DETC function.

Studies of animals with disruption of the Vγ3 gene provided the first evidence that TCR conformation was essential for localization to, and residence in, the skin (21). The epidermis of mice lacking the Vγ3 gene product is populated by γδ T cells expressing alternate Vγ chains, yet these T cells are still recognized by a Vγ3Vδ1 clonotype-specific monoclonal antibody (21). This demonstrates the requirement of TCR conformation for localization of γδ T cells to the epidermal layer of the skin.

Subsequent studies, disrupting the entire TCRδ locus, demonstrated the functional importance of the γδ TCR to both epidermal homeostasis and wound repair. In these TCRδ-deficient animals, the epidermis is populated by replacement T cells bearing diverse αβ TCRs (22). The lack of true DETC in these animals results in keratinocyte apoptosis due to IGF-1 deficiency (23) and gradual decline in epidermal T cell numbers over time as the atypical αβ T cell population is not maintained in the epidermis (22). Upon damage to the epidermal layer, the αβ T cell population found in the epidermis of TCRδ^−^/^−^animals is unable to mount an efficient response to repair the epidermal damage and facilitate the return to homeostasis. One major defect in these animals is a lack of KGF-1 production (24) by the replacement αβ T cells. This results in reduced keratinocyte proliferation and delayed wound closure. In addition, hyaluronan production is defective, resulting in reduced or delayed recruitment of additional immune cells, such as macrophages, required to facilitate the repair process (25).

Wound repair functions of γδ T cells are not restricted to the epidermis. In the DSS-induced mouse model of colitis, it is possible to analyze both tissue damage and repair in the intestine, and thus the role of γδ T cells in these processes. In this model, the importance of γδ T cells in the intraepithelial compartment of the intestine (γδ IEL) to the repair process is clear, yet once again the ligand for the γδ TCR is unknown. Following DSS treatment, γδ IEL localize to sites of epithelial cell damage and express KGF-1, resulting in vigorous epithelial cell proliferation to repair the damage (26). In the absence of γδ T cells, there is increased severity of DSS-induced damage and a delay in tissue repair due, at least in part, to defective KGF-1 production resulting in severely impaired epithelial cell proliferation (26). Together, studies in skin and intestine highlight the importance of the communication between γδ TCR bearing cells and epithelial cells for homeostatic tissue maintenance as well as repair from epithelial damage. What is becoming increasing clear is that TCR–ligand interactions are not the sole communicators for epithelial γδ T cell interactions with their neighboring epithelial cells.

## Costimulation

Costimulation, integral to effective αβ T cell activation, has not been as clearly defined for γδ T cells. However, recent studies have begun to identify novel molecules, and decipher their costimulatory mechanisms, for epithelial γδ T cells.

Junctional adhesion molecule-like (JAML) is a type I transmembrane glycoprotein found on a variety of effector cells of both the innate and adaptive immune system. Most notably, JAML expression has been demonstrated on neutrophils, monocytes, and memory T cells (27, 28). More recently, JAML was found to be expressed at low levels on epithelial γδ T cells under steady-state conditions and rapidly upregulated upon stimulation (29). *In vitro* assays with isolated epidermal γδ T cells demonstrated a key role for JAML in γδ T cell costimulation (29). Strikingly, this costimulatory function of JAML appears restricted to the epithelial subsets of γδ T cells. Emerging evidence suggests that circulating γδ T cells may too have their own unique set of costimulatory and accessory molecules (30–32).

JAML binds to coxsackie and adenovirus receptor (CAR) (28, 29) expressed on epithelial cells (29). CAR ligation of JAML recruits PI3K to JAML (33) and subsequently costimulates DETC proliferation and cytokine production (29). Of note is that PI3K is also able to mediate costimulatory signals through the prototypic αβ T cell costimulatory molecule, CD28, through a binding motif similar to that found in JAML and another αβ costimulatory receptor, ICOS (34). In the absence of JAML-CAR interactions *in vivo* in the skin, DETC activation in response to wounding is impaired, cytokine responses are diminished and subsequent wound closure is delayed (29). Thus, crosstalk between JAML and CAR is a key component of DETC activation and the wound repair process. The comparable expression of JAML and CAR in the mouse intestine (29), suggests that these molecules may play a parallel role in γδ IEL activation in the intestine. Whether interactions between JAML and CAR are also vital for human skin and intestinal T cell activation and damage repair is still unknown.

In addition to JAML, the C-type lectin-like NKG2D receptor is also expressed on effector cells of both the innate and adaptive immune systems. NKG2D can be found on NK, NKT, γδ, and CD8^+^ T cells and is best characterized as providing activating signals upon ligation to one of its multiple ligands (35–37). In humans, NKG2D ligands include MICA and MICB and members of the ULBP family of molecules, while in the mouse, H60a-c, MULT1, and RAE1 serve as ligands (37–39). The expression of NKG2D ligands is generally low under homeostatic conditions, but can be upregulated by a variety of signals of cellular stress including infection, tumorigenesis, and tissue damage.

In the mouse, epidermal γδ T cells express NKG2D. While ligand engagement of NKG2D activates these DETC (40), it is not yet clear whether this activation signal relies on concomitant TCR signaling or can alone activate DETC. H60c is an NKG2D ligand expressed in the epidermis upon skin damage and on cultured keratinocytes (41). H60c engagement of NKG2D, in the absence of TCR-mediated signals, is unable to activate DETC *in vitro*. Instead, H60c provides a costimulatory signal to DETC through NKG2D (41). Blockade of interactions between H60c and NKG2D impairs KGF production and the wound repair response (42). In contrast, keratinocyte specific upregulation of another NKG2D ligand, RAE1, is able to activate DETC directly without an apparent requirement for simultaneous TCR engagement (43, 44). Whether this difference in TCR requirement could be due to the nature of the damage and thus the nature of the induced ligand, and elicited DETC response, is an intriguing question that remains unanswered.

In human beings, the NKG2D ligands MICA and MICB can be recognized by intestinal epithelial T cells expressing the Vδ1 γδ TCR (45, 46). As expression of MIC in the intestinal epithelium is apparently stress induced, these NKG2D ligands have been proposed to be recognized by Vδ1 γδ T cells in their surveillance for signs of damaged, infected, or transformed intestinal epithelial cells (47). Data suggest that MIC recognition can be either directly through the TCR or via NKG2D and that recognition may in fact be sequential, utilizing both molecules (48). This hypothesis, however, remains to be tested experimentally. In addition, both circulating and intestinal γδ T cells have been shown to recognize lipid antigens bound to CD1d [reviewed in Ref. (49)]. Recently, a previously described MICA binding Vδ1 TCR was also found to interact with high affinity with CD1d-sulfatide (50), opening the possibility of multiple ligands for some γδ TCRs.

## Morphology and Migration

Epidermal γδ T cells develop in the thymus during fetal life and migrate to the epidermis, proliferate locally, and then remain in the epidermis for the life of the animal. These cells are sessile under homeostatic conditions (17, 51), and one of their most unique features is their striking dendritic morphology (11, 12), with basal dendrites being highly motile and immobile dendrites apically oriented, anchoring DETC at keratinocyte tight junctions (17). Adoption of these dendritic features seems to be dictated somewhat by environment, as recent work has shown a similar morphology of CD8^+^ T_RM_ in the epidermis (52, 53). Interestingly, T_RM_ cells form short dendrites and small projections that extend laterally within the epidermis (52, 53), in contrast to the long dendrites of DETC projecting upwards toward the stratum corneum (17), indicating that additional non-microenvironmental cues may control epidermal T cell morphology. At least for the γδ T cells in the skin, this dendritic morphology is dramatically lost upon activation of DETC (24). Activated DETC assume a rounded shape and recent studies have identified the semaphorin, CD100, and one of its ligands, Plexin B2, in regulating this process (54). Mice deficient in the CD100 molecule were found to exhibit delayed DETC rounding upon wounding. A direct role for CD100 and plexin B2 in this morphology change was demonstrated by *in vitro* ligation of CD100 leading to ERK kinase and cofilin activation, concurrent with rapid DETC rounding. The importance of the CD100-plexin B2 mediated rounding in epithelial wound repair was demonstrated by the delayed wound closure observed in animals deficient for the CD100 molecule (54). Plexin B2 is broadly expressed on many epithelial tissues where CD100-expressing γδ T cells reside, suggesting a more general role for CD100-plexin B2 in epithelial cell–T cell interactions. Indeed, a more severe colitis and a similar delay in repair, is seen in the absence of CD100 in a mouse model of DSS-induced colitis (55).

Despite an increased understanding of the mechanisms controlling the characteristic DETC rounding upon activation, the function of this morphology change remains to be determined. One idea that has been put forth is that rounding is required for motility of DETC, either to the site of damage following a wound (54), or to draining lymph nodes in response to contact hypersensitivity reactions (56). Interestingly, CD103 has been demonstrated to play a role in DETC dendrite attachment to keratinocytes (17) and has recently been shown to be important for retention of T_RM_ in the skin (57). By analogy with this, CD103 is down-regulated upon DETC activation (22), which may thus allow detachment from keratinocytes and movement of the normally sessile DETC. Consistent with this hypothesis is the reduced number of DETC in the epidermis of CD103-deficient animals (58), although it has not been shown definitively that this is due to a lack of retention of DETC in the skin. Other possible explanations for the reduced DETC numbers in these animals are a defect in DETC development or reduced homing of DETC to the epidermis.

Persistence in the epidermis is also reliant on the aryl hydrocarbon receptor (AhR). AhR is expressed by keratinocytes, Langerhans cells, melanocytes, and DETC (59). In the absence of AhR, DETC undergo apparently normal intrathymic development and are able to home to the epidermis (59, 60). However, DETC in AhR^−/−^ animals do not exhibit their normal dendritic morphology (59). They do not extend dendrites to neighboring epithelial cells, instead remaining round. Furthermore, DETC do not take up residence in the epidermis, but steadily decline in number in the first weeks after their initial homing to the tissue (59, 60). Conditional knock-out animals have demonstrated that it is specifically a deficiency in AhR in the DETC themselves that is responsible for the lack of retention in the epidermis (60), possibly as a result of a defect in c-kit interaction with its ligand, stem cell factor caused by the AhR deficiency (59). AhR-deficient DETC may thus be unable to make the necessary contacts with keratinocytes, and possibly Langerhans cells, that are required for maintenance in the epidermal compartment.

A similar loss of intestinal epithelial T cells in the absence of AhR has been described (60). While normal numbers of γδ T cells were found in lymph node, spleen, and thymus, AhR-deficient animals were virtually devoid of small intestinal TCRαβCD8αα and γδ IEL. As in the epidermis, loss of AhR activity was found to be responsible for a lack of maintenance of these cells in the intestine. Additionally, a reduction in AhR ligands or AhR deficiency itself results in increased immunopathology in DSS-induced colitis (60). Although clearly important for epithelial homeostasis, just how AhR signals maintain DETC and IEL at epithelial sites is unknown. In addition, the role of AhR in the activation of these cells during the wound repair process still requires investigation, but likely requires coordinated interactions between resident γδ T cells and their neighboring epithelial cells.

Interestingly, differences exist between epidermal-resident and intestinal-resident γδ T cells. The epithelia in these two tissues are quite distinct with the epidermis containing a stratified epithelial layer and the intestine lined with a single layer epithelium which may account for some of the differences in the features of γδ T cells in these tissues. As mentioned above, DETC are sessile under homeostatic conditions using their multiple dendritic projections to survey multiple neighboring keratinocytes simultaneously (17). In contrast, γδ T cells in the intestine migrate actively within the intraepithelial compartment in the normal steady state (61). In this way, the limited number of γδ IEL are presumably able to surveil the entire intestinal epithelium for signs of damage or disease. Evidence points to occludin expression by IEL as vital to this process (61) but the contribution of other molecules thought to be involved in epithelial γδ T cell migration, such as CD100 and CD103, is unknown at this time.

## Concluding Remarks

**Figure 1:**
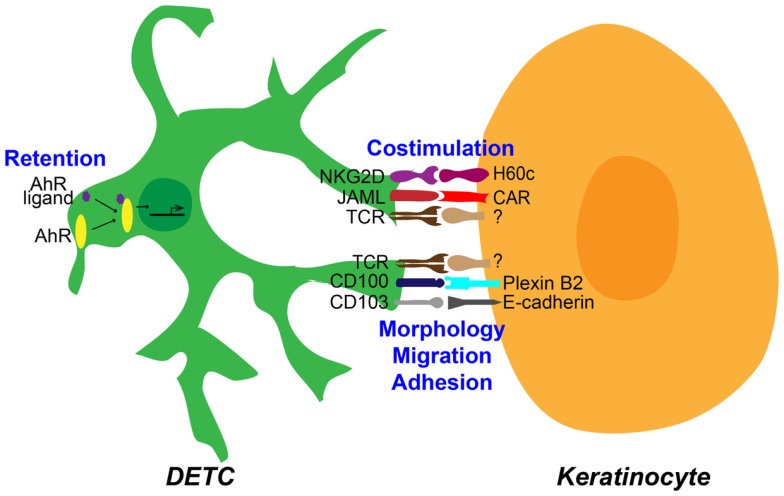
**Epithelial γδ T cell interactions with keratinocytes in the epidermis**. Distinct functional interactions occur between dendritic epidermal T cells (DETC) and neighboring keratinocytes in the epidermis of murine skin. DETC respond to an unknown T cell receptor (TCR) ligand expressed by keratinocytes. This response is concomitant with interactions regulating costimulation, morphology, migration, and adhesion, as well as likely through interactions that mediate retention of DETC in the epidermis. All these interactions are required for efficient DETC activation and effector function.

Although sharing some characteristics with αβ T cells, the identification of an increasing number of novel molecules functioning in various aspects of epithelial γδ T cell activation (Figure 1), highlights the distinct nature of these cells. Numerous molecules, such as integrins, adhesion molecules, cytokine receptors, and known markers of activation are expressed by DETC and other γδ IEL and are modulated *in vitro* and/or *in vivo* by activation signals (62–64). Future studies designed at elucidating the precise role of these various molecules in epithelial γδ T cell activation, should shed further light on the unique functional properties of this enigmatic T cell population.

## Conflict of Interest Statement

The authors declare that the research was conducted in the absence of any commercial or financial relationships that could be construed as a potential conflict of interest.
